# Cryo-Structured Materials Based on Polyvinyl Alcohol and Hydroxyapatite for Osteogenesis

**DOI:** 10.3390/jfb12010018

**Published:** 2021-03-05

**Authors:** Daria N. Lytkina, Dmitriy A. Fedorishin, Polina M. Kalachikova, Anastasiya A. Plyaskina, Aleksandr R. Babeshin, Irina A. Kurzina

**Affiliations:** 1 Chemical Department, National Research Tomsk State University, Lenin 36, 634050 Tomsk, Russia; darya-lytkina@yandex.ru (D.N.L.); strix187@yandex.ru (D.A.F.); Polina.Kalachikova@skoltech.ru (P.M.K.); n08.paa1996@gmail.com (A.A.P.); 2 Skolkovo Institute of Science and Technology, Bolshoy Boulevard 30, bld. 1, 121205 Moscow, Russia; 3 Department of Surgical Diseases with a Course in Traumatology and Orthopedics, Siberian State Medical University, Moskovsky trakt 2, 634055 Tomsk, Russia; alexbabeshin@gmail.com

**Keywords:** cryogel, calcium phosphates, hydroxyapatite, PVA, biocompatible material, bone repair

## Abstract

The application of various materials in biomedical procedures has recently experienced rapid growth. One of the areas is the treatment of many of different types of bone-related diseases and disorders by using biodegradable polymer-ceramic composites. We have developed a material based on cryogel polyvinyl alcohol, mineralized with calcium phosphate. Composites were obtained by cyclic freezing-thawing, the synthesis of calcium phosphates was carried out in situ under the influence of microwave radiation with heating and stirring. The components of the composites were determined using the methods of IR-spectroscopy and scanning electron microscopy and electron probe microanalyzer, as well as their morphology and surface properties. The biological compatibility of the material was investigated in vivo for a Wistar rat. The assessment of the quality of bone formation between the cryogel-based implant and the damaged bone was carried out by computed tomography. An improvement in the consolidation of the bone defect is observed in the bone with the composite in comparison with the control bone.

## 1. Introduction

Bones are capable of self-healing; however, it is common for there to be an external intervention to augment bone repair and regeneration [1]. While traditional methods for repairing bone defects such as autografts, allografts, and xenografts have been widely used, they all have corresponding disadvantages, thus limiting their clinical use [1]. Bone tissue engineering (BTE), a novel approach using scaffolds seeding cells or incorporating bioactive growth factors to promote bone repair and regeneration, is believed to be able to avoid the aforementioned issues and provide an innovative platform in regenerative medicine [2].

Bone is a hard and dense tissue mainly composed of two parts; cortical bone and cancellous, or trabecular bone [3]. The extracellular matrix (ECM) of the bone is a biphasic system, one third of which is composed of organic matter, predominantly type I collagen fibers, and the remaining two thirds consist of inorganic matter or bone salt, such as hydroxyapatite-like calcium phosphates. Three cell types—osteoblasts, osteocytes and osteoclasts—work in concordance to form a unified bone organism. Osteoblasts are the main functional cells of bone formation and are responsible for the synthesis, secretion, and mineralization of bone matrix. They stem from mesenchymal stem cells (MSCs), or progenitor cells, from the adherent portion of bone marrow and cover the surface of bone seams, forming a protein-like mixture called osteoid, which mainly consists of polymerized collagen chains, and is later mineralized into bone, mediated by the deposition of calcium and phosphate. Additionally, osteoblasts also produce the corresponding hormones that promote surrounding bone formation. Osteocytes are inactive post-synthetic osteoblasts that migrated to the ECM matrix of bone. They connect with osteoblasts and other cells and play an important role in mineral homeostasis. The third type of cells, osteoclasts, are derived from hematopoietic stem cells in the non-adherent portion of marrow and are primarily responsible for bone resorption via secreting various matters. The coordinated interactions between osteoblasts and osteoclasts maintain the normal bone mass and aid in the final bone remodeling [4].

Creation of materials for implants providing regeneration of the organism bone tissue is one of the key problems of regenerative medicine. Such materials must contribute to attachment and growth of cells, have appropriate mechanical properties and microstructure similar to the natural bone tissue [5,6].

Hydroxyapatite (HA) is chemically similar to inorganic apatite of the bone matrix with the general formula (Ca_10_(PO_4_)_6_(OH)_2_) [3]. This is one of the most stable modifications of calcium orthophosphate in conditions of the human organism. Owing to this and the absence of toxicity and a negative immune response, it can be applied in biomaterials and implants. The biological activity of hydroxyapatite in physiological conditions (T ≈ 37 °C, pH = 7.4) is related to formation of the calcium-phosphate layer on its surface. Calcium phosphates in such layers are characterized by different structures and compositions since, during precipitation, they interact with ions that are in the body fluids of the organism [7].

Despite the HA advantages, its use in bone implants is limited [8]. This is connected with the low rate of bone resorption in physiologic conditions [9]. The processes of osteogenesis and bone resorption are in equilibrium, and if one of the processes decelerates, then the other decelerates as well. When differentiating osteoblasts, the share of the forming osteoclasts decreases, which results in inhibition of the regenerative processes of organism tissues. The second factor limiting the use of pure HA in materials for regeneration of the bone tissue is its high brittleness and low crack resistance [10]. Brittleness is conditioned by the ionic bond between the atoms in the ceramic material. Ceramic materials are unable to plastically deform; the formation of cracks and breakages is more probable. Resistance of ceramic composites to cracks does not exceed 1.2 MPa·m^−2^, whereas bone strength is from 2 to 12 MPa·m^−2^ [11,12,13,14,15].

Therefore, at present, the studies on the development of composite materials for biomedical purposes are conducted that would combine biological activity and mechanical properties similar to those of the natural bone. Composite HA materials with different polymers allow qualitatively improving mechanical properties of hydroxyapatite [16].

Polyvinyl alcohol (PVA) is biocompatible, available polymer; PVA cryogels are flexible porous materials. When using PVA cryogels in the materials intended for bone implantation, the basic problem is fixation of the material in the organism. On account of biological inertness, PVA shows low adhesion to tissues, which hinders its application [17]. This problem can be eliminated by introducing bioactive and biocompatible components in hydrogel, such as hydroxyapatite. A combination of the cryogel macroporous structure of flexible polyvinyl alcohol and biologically active calcium phosphates must result in a synergetic effect of functional properties of the components of biocompatible composites as bone expletive substances.

The main structural characteristics of the materials strongly depend on the method of production. Therefore, the study of the structure and type of the interaction in the composite material is a relevant task since it allows solving and obtaining materials with the specified characteristics.

Cryogels, a type of polymer scaffold, have several potential advantages in bone repair. Cryogels are composed of three-dimensional hydrophilic polymer chains, which have superior mechanical strength and can provide nutrient environments suitable for endogenous cell growth. They are able to mimic the natural ECM of the bone, thus presenting a prospective ability to encapsulate bioactive molecules or cells. Due to the network structure of the cryogels, the entrapped proteins or cells are confined in the meshes and they cryogels can control the release of the materials as required [18]. Moreover, cryogels are absorbable and demonstrate excellent integration with surrounding tissues, thereby avoiding the complexity of surgical removal and reducing the possibility of an inflammatory response [19]. Additionally, raw materials for preparation of cryogels are extensive and readily available, and they can be tailored to obtain the desired geometry for implantation or injection, and the degradation rate and porosity or release profile can be easily controlled by altering the crosslinking method and degree.

Cryogels based on polyvinyl alcohol have a wide range of potential applications compared to conventional hydrogels due to their macroporous structural network, which ensures efficient mass transfer of dissolved macromolecular substances, ease of access for cell migration, elastic mechanical properties and high biocompatibility [20]. Currently, such cryogels are used as three-dimensional biological scaffolds for various types of cells, including chondrocytes, cardiomyocytes and fibroblasts [21]. The mechanical properties of cryogels can be adapted depending on the intended application by varying the concentration of polymers or crosslinkers, freezing time, temperature and cooling rate [22]. The spongy macroporous system provides a faster kinetic swelling profile and significantly improves viscoelastic properties while preventing physical deformation. In addition, macroporous cryogels provide unhindered transport of solutes, as well as facilitation of cell infiltration, which makes such materials extremely promising for use as bone implants [23]. In addition, the size of macropores present in cryogels can vary from a few micrometers to hundreds of micrometers in diameter, which allows them to accommodate a large number of fillers, including hydroxyapatite powders [24].

Consequently, the development and creation of new cryogels is now becoming an important and promising alternative to traditional bioengineering approaches to tissue regeneration [22].

The purpose of the work is development of new methods of producing cryogel composites of polyvinyl alcohol/hydroxyapatite and study of their physical and chemical properties and in vivo research.

## 2. Results and Discussion

Type A composites were obtained by mixing hydroxyapatite with a PVA solution and subsequent freeze-thaw. Type B composites were obtained by mineralization of a PVA solution in the process of obtaining HA. Formation of the composite materials is determined by the noncovalent interaction between the components in the material volume. A strong intermolecular interaction is typical of polyvinyl alcohol, conditioned by electrostatic attraction of polar hydroxyl substitutes, which are able to orient on the surface of calcium phosphate crystals in the area of Ca^2+^ ions localization. The surface of crystalline calcium phosphates is a phase boundary “calcium phosphate—polymer”; a partial positive charge was compensated on it on account of crystal-lattice defects of the crystals. Lattice defects are determined by the vacancies in the atomic packing. Negatively charged hydroxylic substitutes are attracted to the crystals surface by means of the electrostatic interaction, which determines mutual fixation of the components in the material volume.

**Figure 1:**
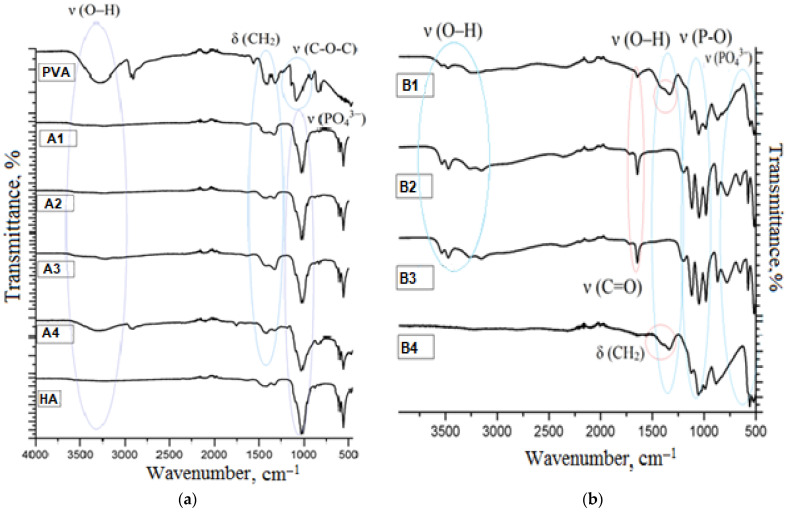
IR-spectra (**a**) composites obtained by method **A**, (**b**) composites obtained by method **B**.

The chemical composition of the materials was studied by IR spectroscopy. Transmittance spectra of the materials, obtained by means of the described methods, are shown in Figure 1.

In the IR-spectrum of pure polyvinyl alcohol there are absorption bands in the range of 1450–1475 cm^−1^ and 770 cm^−1^, which belong to bending deformations of methylene groups in the polymeric chain. Absorption at 2850 cm^−1^ belongs to bond stretches C–H. There is absorption in the range of 1125–1200 cm^−1^ typical of bond stretches C–O, as well as a broad absorption band in the range of 3100–3500 cm^−1^. The latter is a characteristic band for alcohols. It indicates bond stretches O–H. The band is shifted to a low-frequency region on account of the presence of hydrogen bonds between hydroxyl groups in the PVA sample under study. In the hydroxyapatite spectrum there is an intensive absorption band in the range of 1030–1080 cm^−1^, which belongs to stretching bonds O–P–O, but bands of 570–600 cm^−1^ are related to their bending vibrations. The weak absorption band at 962 cm^−1^ characterizes stretching vibrations of the PO_4_^3−^ group. 

The analysis of the obtained composites showed that in the composite spectra, there were bands that were typical of both PVA and HA. For the composites obtained by method A, there are absorption bands typical of pure hydroxyapatite, as well as broad bands of weak intensity of bending vibrations of CH_2_-groups in the range of 1250–1400 cm^−1^. The –OH absorption band of the PVA group is observed only for composite A1 with 50 mass.% of PVA. The composites obtained by method B, have different transmittance spectra. Triple intensive bands in the range of 1000–1080 cm^−1^, corresponding to vibrations of phosphate groups, as well as broad bands from 3500 to 2250 cm^−1^, are typical of them. They represent associated absorption bands of hydro- and dihydrogen phosphate ions. Shifts and unique absorption bands were not found. This is evidence of the fact that formation of new chemical bonds between components does not occur in any of the methods. Crystals of calcium phosphates are retained on the surface of the polymer due to van der Waals interactions. 

**Figure 2:**
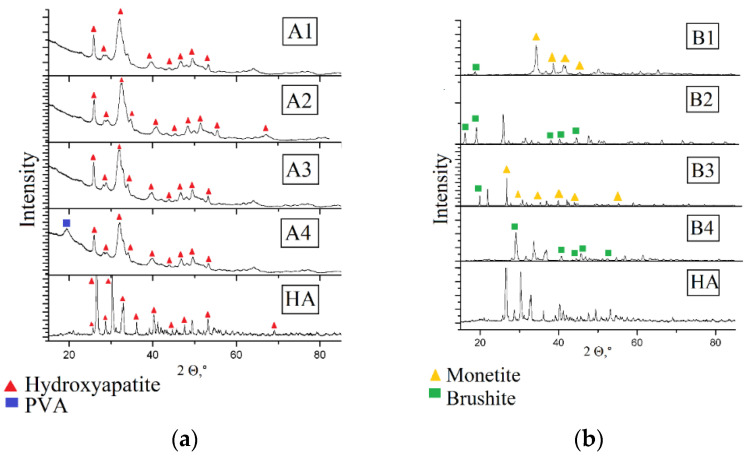
X-ray pattern of composites (**a**) composites obtained by method **A**, (**b**) composites obtained by method **B**.

X-ray diffraction studies are necessary for establishing the structure of calcium phosphates, preliminary synthesized for obtaining the composite. Mechanical properties of the material and its bioactivity depend on the phase composition of the mineral component [15,16]. Figure 2 show the results of the X-ray phase analysis of the obtained composites.

For the composite materials PVA and HA obtained by adding hydroxyapatite the diffraction pattern retains. The obtained composites are amorphous, in composite A4 there is a peak of the PVA crystalline phase corresponding to the parameters of the crystalline lattice a = 1; b = 0; c = 1.

In the composites, obtained by mineralization of the polymeric matrix in situ, the hydroxyapatite phase is absent. Composites B1 and B3 consist of monetite, and composites B2 and B4—of brushite. The materials have a high degree of crystallinity, which, as well as in the case of composites A1–A4, is not influenced by the polymer content in the sample. Monetite and brushite are acid salts of orthophosphoric acid. The triclinic crystal system (space group P1) with the parameters of the lattice: a = 6.916; b = 6.619; c = 6.946 α = 96.18; β = 103.82; γ = 88.34, is typical of monetite crystals. Brushite crystallizes in the monoclinic crystal system; the space group is La; lattice parameters: a = 5.812; b = 15.180; c = 6.239, β = 116.25. Formation of crystals of irregular shape from the solution is observed already at neutral values of pH, which is evidence of violations in the method of hydroxyapatite synthesis. However, it is worth noting that both monetite and brushite are applied in biomedicine: based on them, bone cements and coatings for metallic implants are produced. In physiological conditions both types of phosphates are hydrolyzed with the formation of macroporous carbon-substituted hydroxyapatite, which is characterized by a slower rate of resorption, as compared to hydrophosphates, and acts as a fixing agent.

**Figure 3:**
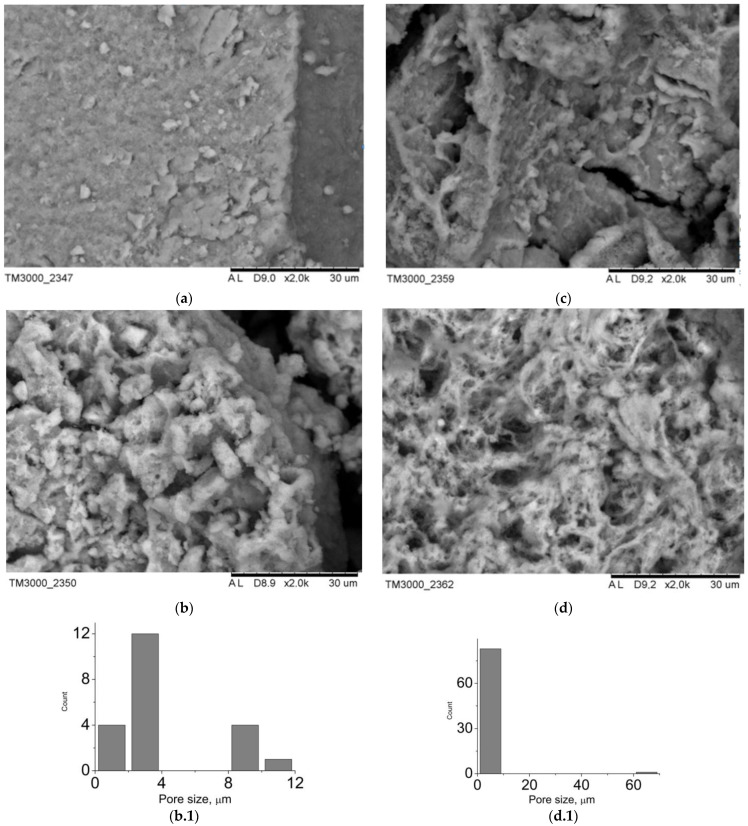
SEM-images of the materials surface: (**a**) A1, (**b**) A2, (**c**) A3, (**d**) A4, pore size distribution: (**b.1**) A2, (**d.1**) A4.

The data of the scanning electron microscopy confirm the results of X-ray phase analysis. SEM images of the composite surface are presented in Figure 3. Composites B1–B4 are characterized by a greater degree of crystallinity. Among the plate crystals of calcium hydrophosphates, white granules of polyvinyl alcohol can be identified. Since the settling of monetite and brushite crystals proceeds in the acid medium, the polymeric matrix collapses: polyvinyl alcohol molecules form isolated aggregates unable to form a three-dimensional network even after several cycles of freeze-thawing. At the same time, in composites A1–A4, there is a transition from polymer, distributed over the surface of the crystals (sample A1), to hydroxyapatite, distributed inside of the macroporous polymeric gel (sample A4). The porosity of the analyzed samples can be judged by the images of the surface: composites B1–B4 have closed porosity, whereas the porosity of composites A1–A4 is open; at the same time mesopores and macropores are present.

**Figure 4:**
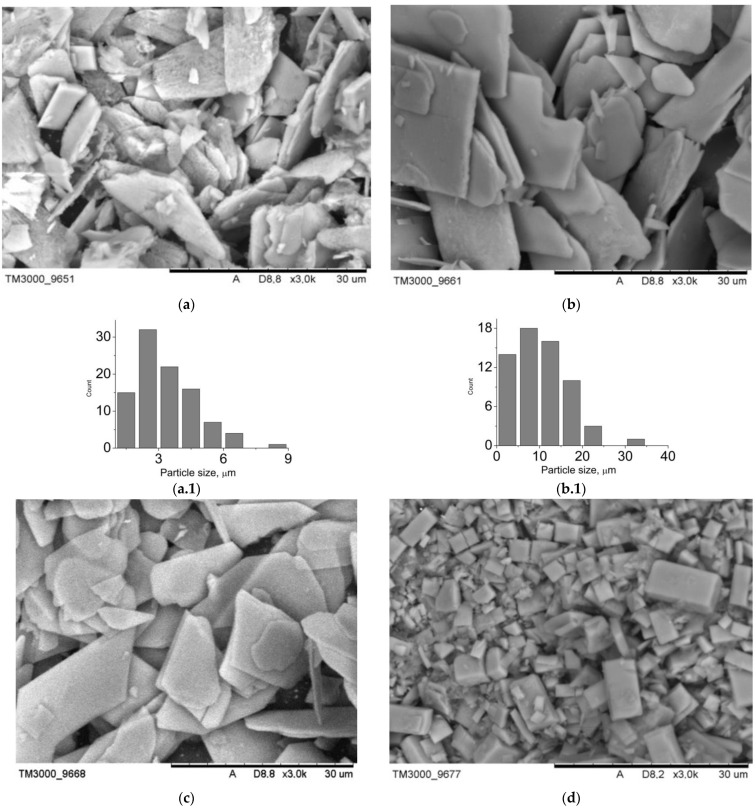

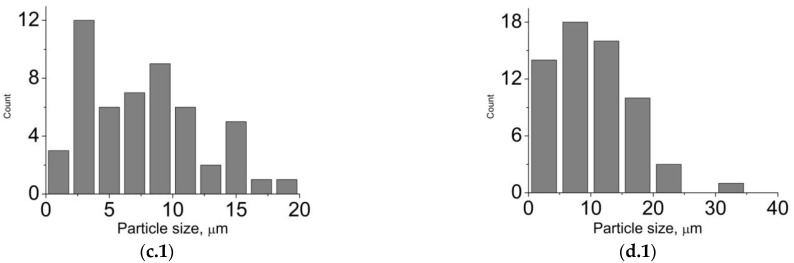
SEM-images of the materials surface: (**a**) B1, (**b**) B2, (**c**) B3, (**d**) B4; particle size distribution: (**a.1**) B1, (**b.1**) B2, (**c.1**) B3, (**d.1**) B4.

The type and the size of the materials pores determine their use in implants. Thus, macropores and mesopores are typical of composite A1 (Figure 3). The presence of pores, exceeding 100 nm, is conditioned by mechanical stirring of the mixture during synthesis. For the rest of the materials, the porosity is in the wide interval from 1 to 25 microns. In case of the composites, obtained by mechanical addition of HA, the porosity is determined by a three-dimensional structure of cryogel. As for composites B1–B4, the porosity is also conditioned by mechanical stirring of the system during synthesis (Figure 4). The shape of particles in composites B1–B4 is determined by their phase composition. Composites, which include the main phase monetite, particles in the form of irregular plates up to 40 μm in size are more characteristic (Figure 4a–c). For sample B4, which is dominated by brushite, cubic particles with a particle size of up to 35 μm are characteristic (Figure 4d).

Based on the obtained physical and chemical methods, sample A4 was chosen, as it showed appropriate porous characteristics best of all; therefore, this sample was chosen for biological studies.

Biological compatibility of the composite materials of hydroxyapatite and cryogel of polyvinyl alcohol was researched using the composite of the A4 composition. The method and the ratio of the components were chosen taking into account established bioactivity of hydroxyapatite and a possibility to fix the material in the area of the bone defect. When the mass fraction of calcium phosphate in the composite increases, the latter becomes more flexible and is able to retain the required shape, but being in contact with liquid, it swells and fills in the available volume.

In the postoperative period, the experimental animals showed a pronounced hypodynamic reaction during the first two days. At the same time, the rats were inactive; when trying to move, they used only the front limbs. Some edema of the operated areas was also noted. In the next five days, these phenomena decreased. At the same time, some edema of the soft tissues of the operated limbs was observed. In the further postoperative period, complete recovery occurred. No complications were found in both groups.

**Figure 5:**
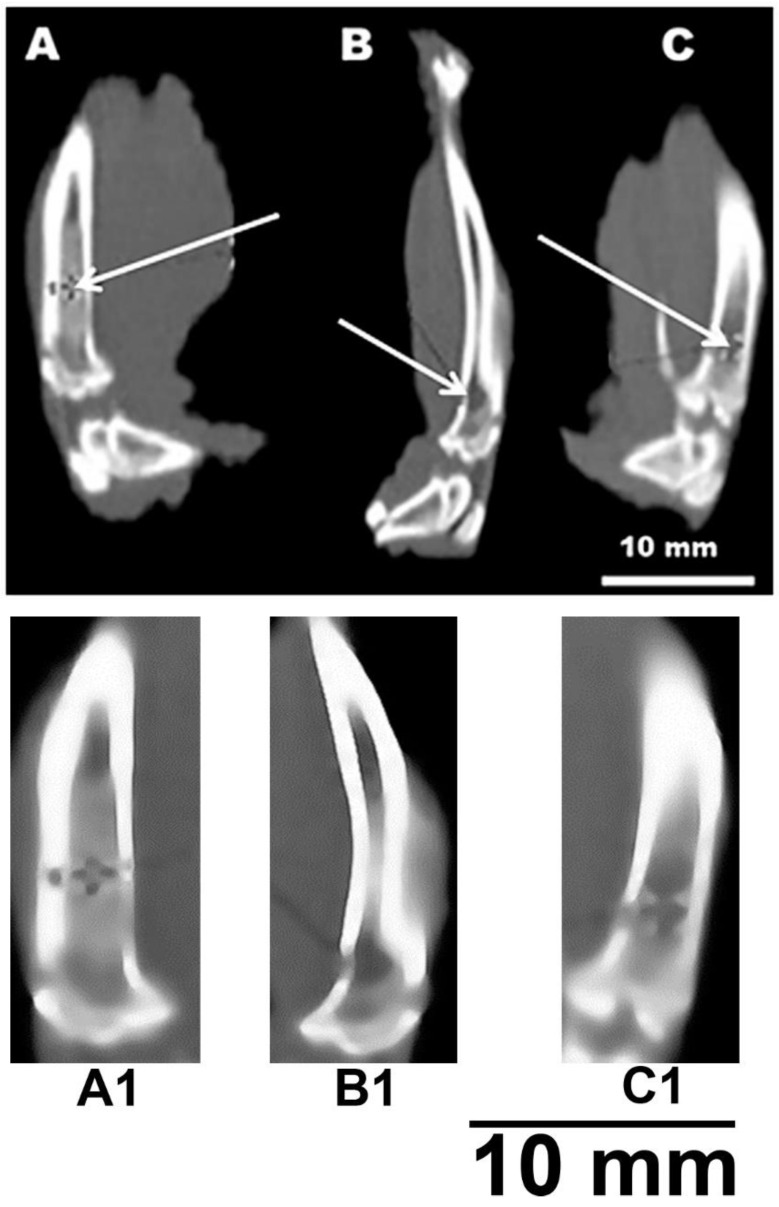
CT image of a rat tibia defect. (**A**,**A1**)—channel with a sample; (**B**,**B1**)—channel without sample (positive control); (**C**,**C1**)—intact bone. White arrows indicate bone defects (**A**,**B**) or intact intramedullary canal (**C**).

Figure 5 shows a fragment of a computed tomogram of the examined bone defects. Tomography was performed on the 80th day of the experiment. Figure 5A shows a partially consolidated fracture of the cortical bone visualized in the bone with a composite at the level of the proximal shaft. In this case, the bone density at the level of the defect is on average +674 HU (Hounsfield units) (*p* < 0.01). In the cavity of the intramedullary canal, a nebulous body made of a composite with an average density of +500 HU was revealed; inlet diameter up to 3 mm (*p* < 0.05). The defect is partially consolidated. The diameter of the bone tissue defect in the experimental group averages 2.1 mm and has a density of +344 HU at the level of the proximal diaphysis (*p* < 0.05). The diameter of the bone tissue defect in the positive control group averages 2.7 mm and has a density of +218 HU at the level of the proximal shaft (*p* < 0.05). The cancellous bone density of the positive control intramedullary canal averaged 84 HU (Figure 5B). The density of the cortical layer of the intact bone averaged +1415 HU (Figure 5C) (*p* < 0.05). The change in the density of the damaged bone makes it possible to determine the biocompatibility of the implant. 

It can be noted that the computed tomography data showed active processes of bone tissue regeneration in the case of using material to fill the defect compared with the control group. In densitometric analysis, the absolute density values in the area of the defect were obtained. According to the literature data, the density of the cortical layer of the intact tibia in rats, on average, is in the range of +1100–+1500 HU. The density of the intermediate zone of the regenerated tissue at the level of the proximal diaphysis is normally +300–+400 HU [23]. At the same time, a similar parameter in the experimental group averages +674 HU. This suggests that the partial consolidation and high density of bone tissue in animals of the experimental group at the level of the proximal diaphysis indicates an acceleration of the healing process of bone tissue after injury. Bone density values suggest that bone healing in the presence of the material is 30% more effective than in the positive control. This observation suggests the possibility of using the materials as a means of bone reconstruction. This also correlates with the statement that gel materials based on PVA and calcium phosphate hold great promise in the treatment of defects in supporting bone [1,7,19].

**Figure 6:**
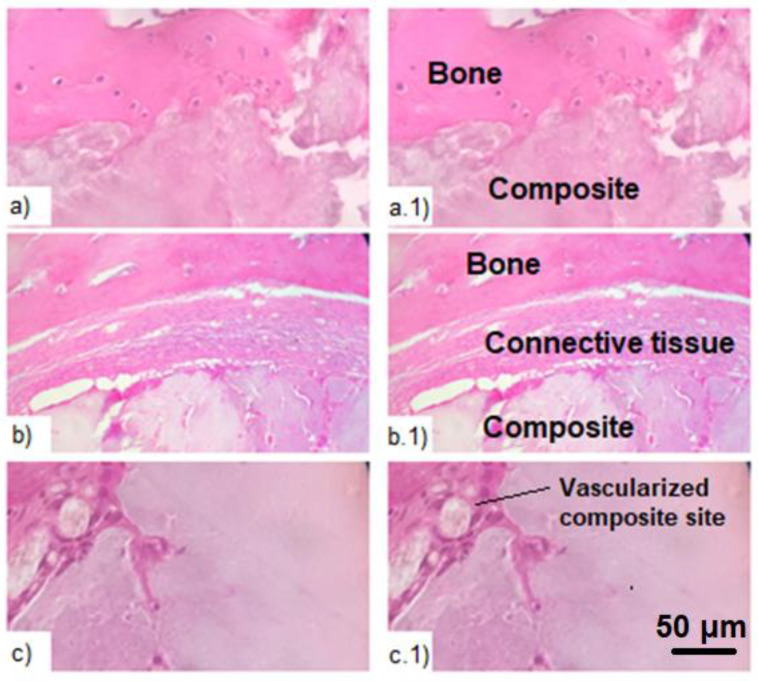
Histologic study of the bone tissue with the introduced composite after hematoxylin-eosin staining. (**a**,**a.1**)—proliferation of osteocytes in the area of the bone defect; (**b**,**b.1**)—connective tissue in the area of defect between the composite and the bone; (**c**,**c.1**)—vascularization of the composite in the area of defect.

Histological studies complement the results of computed tomography, confirming the biological activity of the composite. It was found that in the area of the bone defect there is an overgrowth of osteocytes and the formation of a capsule from the connective tissue between the bone and the composite. At the same time, the connective tissue consists mainly of collagen fibers, macrophages and plasmocytes (Figure 6). At the same time, no pronounced fibrosis was found in the experimental and control groups. Additionally, at the sites of implantation, a local acute inflammatory reaction of moderate intensity and an intensive process of vascularization were revealed. Vascularization promotes the proliferation of osteocytes and enables the formation of new bone tissue. In the experimental group, these processes were somewhat more pronounced than in the positive control group. This may indicate active healing processes at the sites of implant placement and their biological activity.

It should be especially noted that all experimental animals showed no traces of systemic toxic damage to organs during autopsy. Based on this, it can be noted that the acute inflammatory reaction is predominantly local in nature and does not cause systemic damage to the body.

No significant reactions of rejection of implants in animals of the experimental group were revealed. Based on the CT data, histological examination, and animal necropsy, it can be concluded that the studied samples are highly biocompatible.

**Figure 7:**
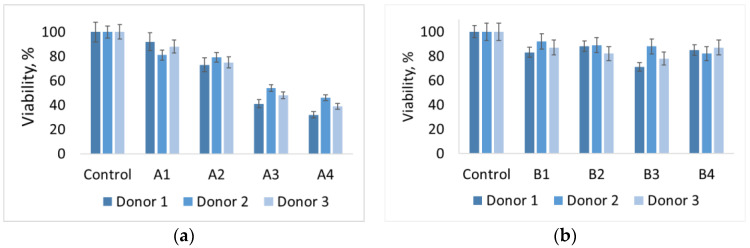
Histogram of the viability level of human macrophages in the presence of materials (**а**) A1–A4 and (**b**) B1-B4, respectively.

Monocytes incubated with A materials, which contain hydroxyapatite, show low viability, and the dependence is inverse to the HA concentration, which suggests precisely the effect of HA on viability—with an increase in its concentration, viability decreases. Materials B containing other phosphates (monetite and brushite) show a similar viability level of 70–90% relative to control. No concentration patterns were found for these materials. The data are shown in Figure 7. Previous studies with hydroxyapatite-based materials [21], we know that macrophages exhibit low viability in the presence of pure hydroxyapatite. However, its use as a component of materials together with polylactide has shown good results. From this we can conclude that polyvinyl alcohol, as a matrix of the composite, is unable to suppress the cytotoxicity of HA in the material. This may be due to the accessibility of the surface of HA particles in the material for cells.

Currently, there is a huge need for materials for the regeneration of bone defects caused by trauma, osteosarcoma, osteoporosis, etc. [25]. As a result of this study, it was found that the composite materials created by the authors on the basis of PVA and hydroxyapatite meet certain criteria for biocompatible materials. These criteria are biocompatibility, biodegradability, osteoconductivity, osteoinductivity, and interconnected porous structure [26]. The interconnected porous structure of the applied materials provides infiltration, migration and proliferation of cells in the place of bone defects. Additionally, similar structure promotes diffusion of nutrients, oxygen and products of metabolism [27,28]. As a result of the study, it was found that the bone density in rats of the experimental group at the level of the proximal diaphysis is significantly higher than in the control group. The healing processes of bone defects under the influence of the composite sample occurred faster than in the control group. According to the literature data, the components included in the composition of the composite are the most optimal for use in the osteoconductive material—the hydrogel on the basis of PVA provides the necessary porosity and biodegradability, and hydroxyapatite facilitates mineralization of the implant. Additionally, this composition of composites was chosen because calcium phosphates themselves do not have osteoinductive properties [27]. Meanwhile, calcium phosphates are widely known for their ability to modulate and complement the extracellular concentration of calcium and phosphate ions. These ions promote proliferation and osteogenic differentiation of progenitor cells as well as MSCs [29]. Cryogel serves as a matrix for hydroxyapatite and provides a slowdown of the output of active ions and reduction of cytotoxicity [28]. We can suppose that this phenomenon can be the cause of the fact that the observed acute inflammatory reaction in the place of implants placement did not reach the pathological level and did not become necrotic. It should be noted that the implants did not cause systemic effects of the lesions in the experimental animals. Their action had a strictly local character.

Tissue engineering strategies that combine matrix-secreting cells with a functional supporting scaffold offer a promising alternative to autologous bone grafts for repairing bone defects. The rat bone defect model is a self-healing defect and assesses the ability of cryogel to accelerate bone repair compared to the natural healing process [30].

Most polymeric cryogels do not have sufficient biological activity to facilitate tissue regeneration. Therefore, at present, research is being actively carried out on composites based on polymer cryogels [31]. Therefore, at present, composite materials based on cryogels are being actively developed. Recently, there has been a report on the use of three-dimensional cryogel in vivo in a defect of the rat bone, which has shown significant prospects for bone regeneration, comparable to autologous bone grafts [32].

To create composites based on polymer cryogels, a variety of fillers are used to give the composites the required properties. According to the literature, one of these fillers is synthetic hydroxyapatite. This material significantly increases the biocompatibility and bioresorbability of the obtained cryogel scaffodes [33].

All animals in the experimental and control groups with sham surgery survived the operation and the postoperative period without complications. After the animals were withdrawn from the experiment as a result of necropsy, none of the animals showed clear signs of perimplant infection or toxic organ damage. Inspection of the implantation sites revealed the encapsulation of cryogel structures by the surrounding tissues. Moreover, the tissue surrounding and encapsulating the cryogel was macroscopically healthy and did not show signs of infection, inflammation, or pathological vascularization. Additionally, all experimental animals showed no signs of toxicity or inflammatory response, which was confirmed histologically. This indicates a high biocompatibility of the material used in the study. It has been experimentally established that the applied sample of the composite can significantly facilitate the natural healing processes of the bone defect. The results obtained during the study are consistent with the literature [30].

As a result of this study, it was found that the applied cryogel-based material is biocompatible and able to integrate with the surrounding bone tissue. According to the literature, such materials can fill micro and macro cracks in the bone, which increases their biocompatibility. The material developed in the course of the study can potentially provide safe and effective bone regeneration for the treatment of small bone defects in cases of nonunion, as well as in clinical cases of osteoporosis and fixation of bone implants.

## 3. Materials and Methods

### 3.1. Materials

Polyvinyl alcohol (PVA) is of M1799 grade (Hongkong XinRunde Chemical Co., Ltd, Hongkong, China), the degree of hydrolysis is 98.0–99.5%; calcium nitrate tetrahydrate—Ca(NO_3_)_2_·4H_2_O (Vecton, Novosibirsk, Russia), ammonium hydrophosphate—(NH_4_)_2_HPO_4_ (Vecton, Novosibirsk, Russia).

### 3.2. Obtainment of Polyvinyl Alcohol Cryogels 

To form two-component cryogels (PVA/water), the PVA aqueous solution in different concentrations was poured into metal cells and frozen at T = −20 °C during 20 h. The obtained ice samples were defrosted for 4 h at room temperature (T ~25 °C). This cycle was repeated 1–3 times. As a result of cryogenic action, flexible and elastic cryogels were obtained from liquid media (polymer solutions). 

### 3.3. Composites Production

Hydroxyapatite was synthesized by the liquid-phase method using microwave radiation at pH~11 according to the scheme [33,34,35,36]:

10Ca(NO_3_)_2_ + 6(NH_4_)_2_HPO_4_ + 8NH_4_OH → Ca_10_(PO_4_)_6_(OH)_2_ + 20NH_4_NO_3_ + 6H_2_O

Initial solutions of calcium nitrate tetrahydrate and ammonium hydrophosphate were poured and stirred on a magnetic mixer; then, they were exposed to microwave radiation. A household microwave oven was used as a microwave reactor; the synthesis was carried out at a power of 100 W until the suspension boils (about 30 min). The obtained precipitate was settled in the mother liquor. Then it was filtered under vacuum and dried at room temperature within 24 h [35,36].

Composite materials were obtained by two methods:

Method A (Table 1). Hydroxyapatite powder was added to a 10% aqueous solution of polyvinyl alcohol during mechanical stirring at a rate of 400 rev./min. The suspension was stirred for 4 h. After stirring, the suspension was transferred to test tubes Falcon for freeze-thawing. Three cycles of freeze-thawing were conducted the continuation of each stage was 24 h. The freezing temperature was −80 °C, the thawing temperature was +25 °C.

Method B (Table 1). The 10% aqueous solution of polyvinyl alcohol was added to the aqueous solution of calcium nitrate Ca(NO_3_)_2_. During heating, the aqueous solution of ammonium hydrophosphate (NH_4_)_2_HPO_4_ was added to the obtained mixture. The resulting suspension was heated during stirring (T = 50 °C) for 8 h and cooled to room temperature. Later there were three cycles of freeze-thawing in conditions described in method A.

### 3.4. Physical and Chemical Research Methods

X-ray phase analysis of the composites of hydroxyapatite and polyvinyl alcohol was conducted on the diffractometer Rigaku Smartlab (CuKα—radiation Rigaku Corporation, Tokyo, Japan) by the powder method. The phase composition was determined using the COD databases of Cambridge University and ICDD, card 010738418. IR-spectra of the obtained composite materials were photographed using the IR-Fourier spectrometer Agilent Cary 630 (Agilent Technologies Inc., Santa Clara, CA, USA). The surface morphology of the composite materials of hydroxyapatite and polyvinyl alcohol was studied on the electron microscope HITACHI TM-3000 (Hitachi High-Tech Corporation, Tokyo, Japan) under the acceleration voltage of 15 kV. The attachment Quantax-70 (Bruker Corporation, Billerica, MA, USA) was used for elemental energy dispersive microanalysis. The parameters of the porous structure and specific surface of the composite materials of hydroxyapatite and polyvinyl alcohol were assessed using the gas-adsorptive analyzer TriStar II (Micromeritics Instrument Corporation, Norcross, GA, USA). The degassing conditions were 2 h, temperature—130 °C, pressure—1 atm.

### 3.5. Assessment of Biocompatibility of Materials In Vivo

An in vivo experiment was performed to assess the rate of tissue formation between the implant and the damaged bone. The test system for the experiment was male Wistar rats (*Rattus norvegicus forma alba*) 8 weeks old and with an average weight of 200–240 g (*n* = 20). All rats were divided into two equal groups—experimental and control. The size of each group was 10 animals. The sample size was calculated using the resource method [37].

A composite consisting of PVA hydrogel and unmodified hydroxyapatite in a 1: 1 ratio was used for the study. The samples were rods 3 mm in diameter and 5 mm in height. Each implantable sample was fabricated, cleaned and sterilized in accordance with the technology used for bone implants. All implant manipulations were performed under aseptic conditions to prevent damage or contamination of the samples before or during implantation.

Bone defects in experimental animals were created in the proximal metaphyses of the tibia. The anteromedial approach to the tibia was used for the operation. Bone defects were intramedullary channels with a diameter of 3 mm and a depth of 5 mm, drilled with a diamond drill. In animals of the experimental group, two bone defects were created in both tibia. These defects were filled with the studied composite samples. The samples were introduced into the channels using a syringe with a thick blunt needle, while the sample was pushed into the formed channel from the needle using a mandrel. In animals of the control group, a canal was drilled in the right tibia, similar in size to that in the experimental group. The left bone remained intact. After the operation, the wounds were sutured in layers with Vikril (copolymer of lactide and glycolide) and treated with an antiseptic. A 0.5% alcoholic solution of chlorhexidine was used as an antiseptic.

After withdrawal from the experiment, the animals were subjected to humane euthanasia followed by complete necropsy. In this case, the implants were cut out with a sufficient amount of surrounding bone tissue for further histological examination. Tissue samples from experimental animals taken from the implantation sites were fixed using a buffered formalin solution according to the standard protocol. Decalcification of the obtained bone material was carried out by an acid-free method using 10% EDTA (pH 7.4) at 4 °C for 5 days. After decalcification, the samples were subjected to dehydration in a series of alcohols with increasing concentrations—70%, 80%, 96%, and 100%.

After fixation and decalcification, tissue samples were frozen and cut on a Thermo Scientific Microm HM 525 cryotome (Thermo Fisher Scientific Inc., Waltham, MA, USA) at −22 °C. The sections were 50 μm thick. After cutting, the samples were subjected to hematoxylin-eosin staining according to the standard protocol. Microscopic observation of tissue samples was performed using a Zeiss AxioImager Z2 microscope (Carl Zeiss Microscopy GmbH, Jena, Germany).

### 3.6. Statistical Analysis

Statistical analysis was performed using Microsoft Office Excel and StatSoft STATISTICA 10.0 for Windows (StatSoft Inc., Tulsa, OK, USA). Normal distribution was tested using the Shapiro–Wilk test. The Mann–Whitney U-test was applied to analyze differences in the differences in Hounsfield units. Differences were considered significant at *p* < 0.05. Differences at 0.05 > *p* < 0.1 were discussed as non-significant trends.

### 3.7. Keeping, Care and Using of Laboratory Animals

Surgical interventions were made using inhalation anesthesia by means of the anesthesia machine Combi Vet. The anaesthesia was induced by gas “Sevoran”, an active component of which was sevoflurane. To introduce general anaesthesia, inhalation was applied using sevoflurane in the concentration of 8%. To maintain the anaesthetic effect, inhalation proceeds with the decrease in the sevoflurane concentration to 0.5–3%. The state of anesthesia in animals verified by the disappearance of the reaction to painful stimuli (prick of the paw) and inhibition of the corneal reflex. The duration of the experiment was 80 days. There were no cases of unplanned death of animals during the experiment.

The experimental animals were kept in a specially equipped conventional type vivarium with free access to water and food, and a 12/12 light regime. Keeping animals and setting up experiments was carried out in accordance with the guidelines of the Declaration of Helsinki, and international rules “Guide for the Care and Use of Laboratory Animals”, as well as GOST 33216-2014 “Guidelines for the maintenance and care of laboratory animals. Rules for the maintenance and care of laboratory rodents and rabbits” and approved by the Ethics Committee of Tomsk State University (Protocol of the meeting of NR TSU Bioethics Commission, Protocol № 2/1 from 23 March 2019, extract from Protocol № 2). After removal from the experiment, the animals were subjected to humane euthanasia in accordance with international rules. Euthanasia was carried out in accordance with GOST 33216-2014 by inhalation of 100% carbon dioxide (CO_2_). All the experimental manipulations with animal did not include painful and inflicting suffering procedures and were approved by the Tomsk State University Bioethics Committee. All work with laboratory animals was strictly regulated in accordance with the 3R rules.

### 3.8. Assessment of Biocompatibility of Materials In Vitro

Assessment of the viability of the immune system cells after incubation on the surface of the test materials was performed by the following procedure: during the analysis, monocytes isolated from human blood were first inoculated onto the samples. Monocytes were isolated from the blood of three donors. Cell pellet was resuspended in Macrophage serum- free medium or X-Vivo at a concentration of 1 × 10^6^ cells per mL. Cells were seeded in 12-well plates with samples (2 mL per well). Then, the samples were incubated at 37 °C for 6 days. After that, the supernatant was taken from each well, leaving 500 μL of medium with cells in the well. AlamarBlue reagent (50 μL AlamarBlue/cell medium volume ratio 1/10) was added to the wells. Cells with AlamarBlue were incubated for 3 h at 37 °C in a dark place. After incubation, the cell medium with AlamarBlue was added to a 96-well plate (three wells for each sample). The intensity of the fluorescence signal was measured using a Tecan Infinite 200 microreader (Tecan Group Ltd., Mannedorf, Switzerland) at a wavelength of 540 nm.

## 4. Conclusions

A method of obtaining the composite materials of polyvinyl alcohol and calcium phosphates was developed by adding dispersed hydroxyapatite to the concentrated solution of polyvinyl alcohol and in situ mineralization of the PVA concentrated solution. The obtained composites are characterized by a mass ratio of the components (HA/PVA): 90/10, 95/5, 75/25 and 50/50, respectively.

According to the data of the infrared spectroscopy and X-ray phase analysis, formation of stable chemical bonds between the components in the materials was not detected. Polyvinyl alcohol does not influence significantly the crystalline lattice of hydroxyapatite; however with the significant content of the polymer it crystallizes, which is confirmed by reflexes in the X-ray patterns. When obtaining the composites by in situ mineralization of the PVA solution, formation of the phase of hydroxyapatite, as well as the crystalline phase of polyvinyl alcohol, was not noted. The obtained calcium phosphates are intermediate constituents of hydrolysis of Ca_3_(PO_4_)_2_ up to hydroxyapatite. Principal lines typical of the initial components are present on the spectra. The composites belong to mesoporous materials; the pore size is suitable for vascularization.

In vivo studies confirmed the alleged bioactivity of the composites. Proliferation of osteocytes, formation of the connective tissue in the area of the bone defect between the composite and the damaged bone, as well as vascularization of the composite, are observed. Resorption of the composite proceeds in the intramedullary canal. At the same time, the in vitro study showed that for materials based on cryogels and calcium phosphates, the composition of the phosphate in the material has a significant effect. Varying the composition of the filler leads to a change in the viability of monocytes in the presence of materials. Thus, the presence of hydroxyapatite reduces the viability, while monetite and brushite do not significantly affect the viability.

## Figures and Tables

**Table 1:** Composite materials composition.

Method A			Method B		
Designation	HA, Mass.%	PVA, Mass.%	Designation	PVA, Mass.%	HA, Mass.%
A1	99	1	B1	1	99
A2	95	5	B2	5	95
A3	75	25	B3	25	75
A4	50	50	B4	50	50

## Data Availability

Data sharing is not applicable to this article.
